# Transcriptome Analysis of *Plenodomus tracheiphilus* Infecting Rough Lemon (*Citrus jambhiri* Lush.) Indicates a Multifaceted Strategy during Host Pathogenesis

**DOI:** 10.3390/biology11050761

**Published:** 2022-05-17

**Authors:** Angelo Sicilia, Riccardo Russo, Marco Caruso, Carmen Arlotta, Silvia Di Silvestro, Frederick G. Gmitter, Alessandra Gentile, Elisabetta Nicolosi, Angela Roberta Lo Piero

**Affiliations:** 1 Department of Agriculture, Food and Environment, University of Catania, Via Santa Sofia 98, 95123 Catania, Italy; angelo.sicilia@unict.it (A.S.); gentilea@unict.it (A.G.); enicolo@unict.it (E.N.); 2 Citrus Research and Education Center, Institute of Food and Agricultural Sciences, University of Florida, Lake Alfred, FL 33809, USA; riccardo.russo.1991@gmail.com (R.R.); fgmitter@ufl.edu (F.G.G.J.); 3 Council for Agricultural Research and Economics, Research Centre for Olive, Fruit and Citrus Crops, Corso Savoia 190, 95024 Acireale, Italy; marco.caruso@crea.gov.it (M.C.); arlottacarmen@gmail.com (C.A.); silvia.disilvestro@crea.gov.it (S.D.S.)

**Keywords:** *Plenodomus tracheiphilus*, transcriptome, mal secco disease, fungus RNAseq, *Citrus jambhiri*, rough lemon

## Abstract

**Simple Summary:**

The cultivation of the lemon is strongly impacted by mal secco, a disease that causes huge losses in yield every year. In this work, we have identified, retrieved, and classified genes that may play a crucial role in the onset and progression of the disease. Understanding the function of these genes will increase knowledge of the processes involving the mode of action of necrotrophic fungi during pathogenesis. Our results may be relevant to help identify sustainable field treatments to cope with disease diffusion and to provide direction into possible biotechnological approaches to generate resistant lemon plants.

**Abstract:**

The causal agent of mal secco disease is the fungus *Plenodomus tracheiphilus*, mainly affecting lemon tree survival in the Mediterranean area. Using a fully compatible host-pathogen interaction, the aim of our work was to retrieve the fungus transcriptome by an RNA seq approach during infection of rough lemon (*Citrus jambhiri* Lush.) to identify crucial transcripts for pathogenesis establishment and progression. A total of 2438 clusters belonging to *P. tracheiphilus* were retrieved and classified into the GO and KEGG categories. Transcripts were categorized mainly within the “membrane”, “catalytic activity”, and “primary metabolic process” GO terms. Moreover, most of the transcripts are included in the “ribosome”, “carbon metabolism”, and “oxidative phosphorylation” KEGG categories. By focusing our attention on transcripts with FPKM values higher than the median, we were able to identify four main transcript groups functioning in (a) fungus cell wall remodeling and protection, (b) destroying plant defensive secondary metabolites, (c) optimizing fungus development and pathogenesis, and (d) toxin biosynthesis, thus indicating that a multifaceted strategy to subdue the host was executed.

## 1. Introduction

“Mal secco” disease (MSD) is a serious disease of citrus caused by the mitosporic, necrotrophic fungus *Plenodomus tracheiphilus* [1], which is included in the A2 list of quarantine pests of the European and Mediterranean Plant Protection Organization (EPPO). Although most citrus species are susceptible when artificially inoculated with the fungus [2], lemon (*Citrus limon* L. Osbeck) is one of the most susceptible, and economic losses associated with MSD are dramatic for this species. MSD directly impacts the production volumes, but several indirect impacts are related to the very high costs connected to the disease control (pruning of affected branches and replanting of dead plants) and by using t tolerant cultivars that are characterized by lower fruit quality, thus reducing the economic value of the marketable lemons [3]. The current geographical distribution of MSD comprises the east coast of the Black Sea (Georgia) and all citrus-growing countries of the Mediterranean basin, except Morocco and Portugal (EPPO global database. https://gd.eppo.int/taxon/DEUTTR, accessed on 17 January 2022). The symptomatology of the disease is characterized by the desiccation of twigs, branches, or the whole plant, as suggested by its internationally adopted name “mal secco”, meaning “dry disease” in Italian [4]. The fungus enters the plant through wounds [5,6], and then it enters the vessels and spreads systemically inside the host, colonizing the neighboring xylem tissues [7]. The disease progresses basipetally, more or less rapidly, and finally, the tree meets its death [8,9]. The rate and extent of xylem colonization proved to be directly related to symptom severity that becomes evident mainly in the spring. *P. tracheiphilus* causes leaf vein chlorosis and shoot wilting, followed by leaf dropping, shedding, and dieback of apical twigs [7,8]. A salmon pink or brown-reddish wood discoloration under the bark of withering twigs, infected branches, and the trunk is the most typical symptom of the disease, which is due to the gum production within the xylem vessels [10]. Previous results aimed to discover the relationship between isolate identity and pathogenesis concluded that there is no significant variation in the pathogenicity of *P. tracheiphilus* isolates collected from different Mediterranean countries [7]; even the genetic variability among different populations originating from Italy, Israel, and Greece is very low, this being a direct consequence of the agamic reproduction system adopted by the fungus [11,12,13,14,15]. Based on the analysis of randomly amplified polymorphic DNA (RAPD), microsatellite markers, and sequencing of the internal transcribed spacer (ITS) region of the nuclear rRNA genes, Balmas et al. [11] deduced phylogenetic relationships among Italian isolates of *P. tracheiphilus*, indicating that they are genetically homogeneous, generating identical patterns upon amplification with all primers tested. Accordingly, ITSI-5.8S-ITS2 sequences of all *P. tracheiphilus* isolates were extremely conserved (98–100% identity along a 544 character alignment) [7]. Analogous results were obtained by Ezra et al. [14], who analyzed a small population of the fungus (22 isolates) collected in Israel. The arbitrary primed polymerase chain reaction profiling (apPCR) showed very similar patterns for all the isolates whose homogeneity was confirmed by the comparison of ITS1-5.8S-ITS2 sequences [14]. This genetic homogeneity would indicate that the Israel fungal population perhaps originates from a common ancestor [14]. However, chromogenic and non-chromogenic isolates could be differentiated based on the capability to produce red pigments in culture [16], thus suggesting that a different regulation of gene expression might occur among genetically similar isolates. 

Several studies have been performed to elucidate the secreted proteins, processes, and metabolites involved in the lemon/*P. tracheiphilus* host-pathogen interaction, which can actively concur in disease onset and progression. A key role of enzymes degrading the plant cell wall has been reported, which either intervenes in the disease’s late-stage but not in vessel colonization [7] or induces electrolyte leakage in tissues, as observed in sour orange leaves [17]. Other physiological and biochemical aspects of various pathogenic fungi are involved in necrotrophic pathogenesis, characteristics including the secretion of secondary metabolites and toxins [18]. Glycoproteins of 93 KDa and 60 KDa (called Pt60) belonging to the malseccin complex have been isolated from fungus culture filtrates and host plants infected by *P. tracheiphilus* [19]. In many cases, toxins have been shown to reproduce, in part or in whole, the symptoms of the disease [20,21]. The toxic effects of the malseccin complex on citrus leaves are clearly visible only under illuminated conditions, suggesting that light plays a role in the toxin activity [8]. These findings support the hypothesis that the reduction in photosynthetic rate in citrus plants affected by mal secco is mainly due to metabolic disorders of the photosynthetic process caused by the toxin [8]. Recently, two different approaches have been undertaken to identify potential resistant or tolerant sources to be used in citrus breeding programs [22,23]. An open-field survey on lemon and lemon-like germplasm in response to *P. tracheiphilus* natural infections was performed by visual observation of symptoms and pathogen detection by real-time PCR. These results represented a milestone in the recognition of tolerant lemon accessions, especially in regards to selecting suitable parents for the introgression of resistance genes into lemon genotypes [22]. Moreover, as global transcriptomic analysis has become a powerful tool to discover candidate genes involved in responding to abiotic [24,25] and biotic stress [23], we analyzed the transcriptomic response of rough lemon (*C. jambhiri* Lush.) to *P. tracheiphilus* infection by RNA sequencing and transcriptome de novo assembly [23]. In that work, we dissected the molecular response of a susceptible citrus species to the fungus, which basically implies the activation of the systemic acquired resistance (SAR) through the induced salicylic acid cascade. Interestingly, based on gene expression data, RPM1 interacting protein 4, triggering the plant defense system including the hypersensitive response (HR), was found down-regulated in the inoculated plant, suggesting that it might have a role in the susceptibility of rough lemon, which was no more able to avoid the pathogen circulation inside the plant [23]. In this work, by taking advantage of the aforementioned RNA seq analysis, we unraveled the transcriptional dynamics of *P. tracheiphilus* genes that were commonly expressed during both its establishment and necrotrophic phases of pathogenesis on rough lemon leaves. Several candidate pathogenicity determinants, including putative effectors and their roles during *P. tracheiphilus* pathogenesis, were identified.

## 2. Materials and Methods

### 2.1. Plant Material and Inoculum

Rough lemon (*C. jambhiri*) seeds were sowed on sterile peat in May 2019. After 6 months of growing in a chamber at 25 °C and 90% humidity, the plants were inoculated with the pathogen *P. tracheiphilus* PT10 strain (kindly provided by Professor Vittoria Catara, University of Catania). This chromogenic strain was isolated from symptomatic lemon trees in Sicily [26]. The strain was chosen because it is phenotypically stable in synthetic medium (potato dextrose agar (PDA), carrot agar), exhibits no changes in morphology or virulence, unlike other strains (unpublished data), and shows a medium virulence [15]. 

The inoculum was prepared using a slight modification of the method described by Salerno et al. [27], as detailed in Russo et al. [23]. The inoculation (inoculum concentration at 10^6^ mL^−1^) was performed by depositing 10 µL on wounds obtained by cutting the midvein of three leaves for each plant with a sharp, sterile blade, whereas control plants were inoculated with 10 µL of water. Both were inoculated, and control samples (five plants for each of the treatments) were collected 15 days after inoculation. At that stage, inoculated plants showed evident symptoms of the disease. The leaves were immediately frozen with liquid nitrogen and stored at −80 °C until both DNA and RNA extractions were performed [23].

### 2.2. DNA Extraction and Real-Time Confirmation of Infected Plants

The DNA extraction was performed as described in Russo et al. [23]. The DNA concentration and purity were checked by a NanoDrop 2000 spectrophotometer (Thermo Scientific™, Waltham, MA, USA). Taqman Real-time PCR was performed to reveal the presence of the pathogen within the inoculated plants using an ABI 7500 Real-Time PCR System (Applied Biosystems™, Foster City, CA, USA). The analysis was performed according to the method described in Licciardello et al. [13], using DNA extracted from both inoculated and control leaves as a template.

### 2.3. RNA Extraction and Library Preparation and Sequencing

The RNA was extracted using the RNeasy^®^ Plant Mini Kit (Qiagen, Venlo, Netherlands) according to the manufacturer’s instructions. RNA degradation and contamination were monitored on 1% agarose gels. The RNA purity and concentration were checked using the NanoDrop 2000 spectrophotometer (ThermoFisher Scientific, Waltham, MA, USA). Before sequencing, sample RNA integrity (RIN) was assessed using the Agilent Bioanalyzer 2100 system (Agilent Technologies, Santa Clara, CA, USA) [23]. After the QC procedures, sequencing libraries were generated using NEBNext^®^ Ultra™ RNA Library Prep Kit for Illumina^®^ (New England Biolabs, Ipswich, MA, USA) following the manufacturer’s recommendations and as reported by Russo et al. [23]. After cluster generation (Novogene Bioinformatics Technology Co., Ltd., Beijing, China), the libraries were sequenced on the Illumina HiSeq2000 platform to generate pair-end reads. The clean data were obtained from raw data (raw reads) by removing reads containing adapters, reads containing poly-N, and low-quality reads. Sequences putatively belonging to the pathogen in the inoculated rough lemon samples were recovered by filtering out the reads mapped to the fungus genome (https://mycocosm.jgi.doe.gov/Photr1/Photr1.info.html accessed on 7 February 2022). The validation of the RNA seq experiment was performed by real-time PCR gene expression measurement of selected transcripts [23]. Sequences were uploaded to NCBI (https://www.ncbi.nlm.nih.gov/geo/ accessed on 29 December 2020) accession number GSE164096.

### 2.4. Fungus Transcriptome Recovery

To retrieve the fungus transcripts, 2438 clusters were chosen on the basis of their annotation in the Nucleotide (Nt) database. All the sequences belonging to the pathogen in the inoculated rough lemon samples were validated by mapping the reads to the fungus genome (https://mycocosm.jgi.doe.gov/Photr1/Photr1.info.html accessed on 7 February 2022). Gene function was annotated based on the following databases: National Center for Biotechnology Information (NCBI) non-redundant protein sequences (Nr), NCBI non-redundant nucleotide sequences (Nt), Protein family (Pfam), Clusters of Orthologous Groups of proteins (KOG/COG), Swiss-Prot, Kyoto Encyclopedia of Genes and Genomes (KEGG), Ortholog database (KO), and Gene Ontology (GO). The GO and KEGG annotations were further investigated to obtain a detailed view of the main functions and pathways adopted by the fungus during plant infection. In particular, Unigenes annotated in the KEGG database were grouped according to the KEGG pathway ID. A similar approach was used to determine the main GO terms, divided into Biological Process (BP), Molecular Function (MF), and Cellular Component (CC) involved in fungus establishment and progression. In both cases, categories composed of more than five Unigenes have been considered. All the presented data are based on FPKM (fragments per kilobase of exon model per million reads mapped), which is the most common unit reported to estimate gene expression based on RNAseq data [28]. As a consequence, the analysis concerns the global absolute fungal gene expression during plant colonization at a specific stage (15 days post-inoculation). All of the considered clusters were not in the mock-inoculated control plants. The boxplots of FPKM distribution were developed using the boxplot R function accessed on 7 March 2022. To ameliorate the aspect of the figures the log_10_(FPKM+1) was used instead of FPKM. The Unigenes average FPKM values of the three biological replicates were used as the source dataset, and a threshold of 0.11 [log_10_(FPKM+1) > 0.11] was applied since Unigenes whose log_10_(FPKM+1) was less than 0.11 were considered not expressed (FPKM > 0.3). The outliers, defined as a data point that is located outside the fences (“whiskers”) of the boxplot, were calculated by the boxplot R function and include data points that are 1.5 times outside the interquartile range, above the upper quartile, and below the lower quartile. 

## 3. Results

### 3.1. Analysis of Plenodomus tracheiphilus Infection on Rough Lemon Leaves and Fungus Detection

The effectiveness of fungal inoculation was evaluated by both a visual inspection of the inoculated leaves and by the detection of the fungus genome by Taqman Real-Time PCR. As reported in Russo et al. [23], the typical disease symptoms were detected 15 days after inoculation. The real-time quantitative detection of *P. tracheiphilus* in rough lemon leaf was detected exclusively in inoculated plants (data not shown) [23].

### 3.2. Fungus Transcriptome Recovering and Transcript Functional Annotation

**Figure 1:**
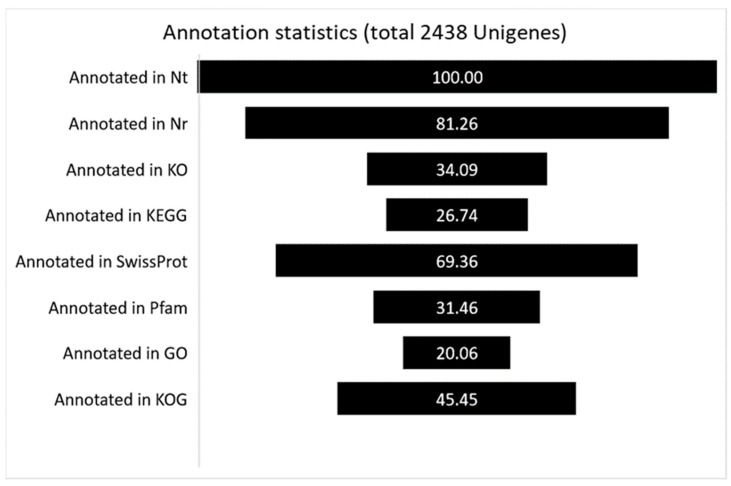
The percentage of successful annotated genes in several databases.

As described in the Section 2, 2438 Unigenes for which Nt annotation could be attributed to *P. tracheiphilus* were retrieved, and their sequences mapped back to the *P. tracheiphilus* genome. To achieve comprehensive gene functional annotation, all of the assembled Unigenes were blasted against public databases, including NCBI, Pfam, KOG/COG, SwissProt, KO, and GO (Figure 1). Among them, 81,26% of assembled Unigenes showed identity with sequences in the Nr. The percentage of assembled Unigenes homologous to sequences in KO, KEGG, Swiss-Prot, Pfam, GO and KOG databases were 34.09, 26,74, 69.36, 31.46, 20.06, and 45.45%, respectively (Figure 1).

### 3.3. Functional Classification of Fungus Transcripts

**Figure 2:**
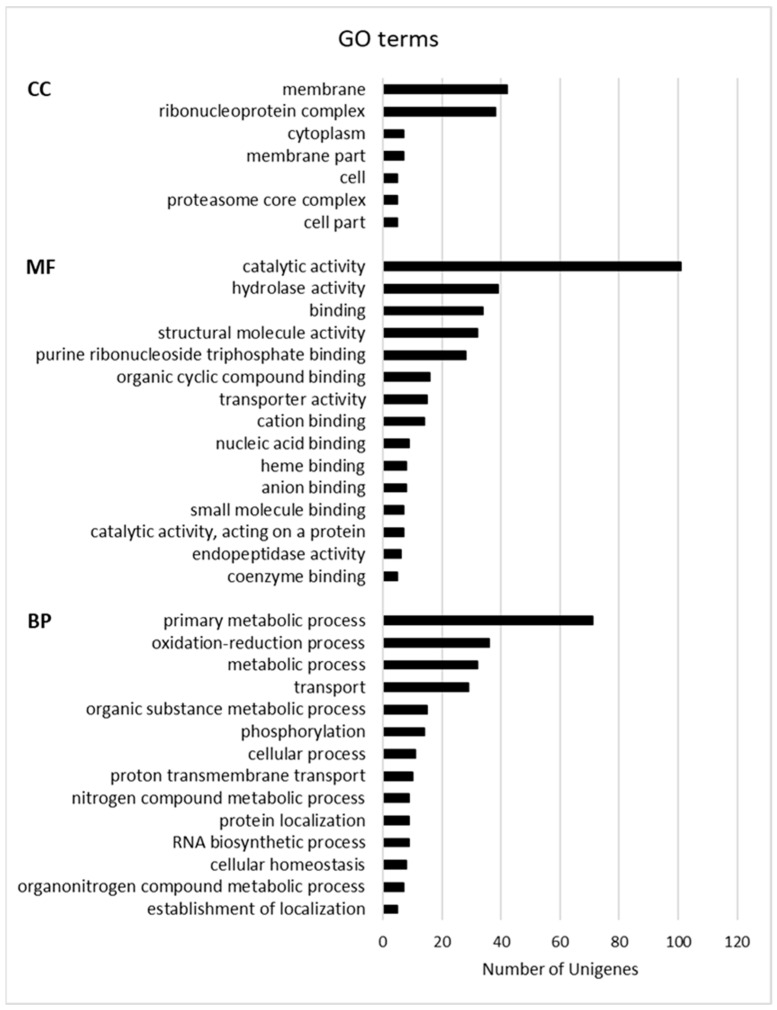
Most represented GO terms. Terms with at least five Unigenes are listed. CC (Cellular Component), MF (Molecular Function), BP (Biological Process).

**Figure 3:**
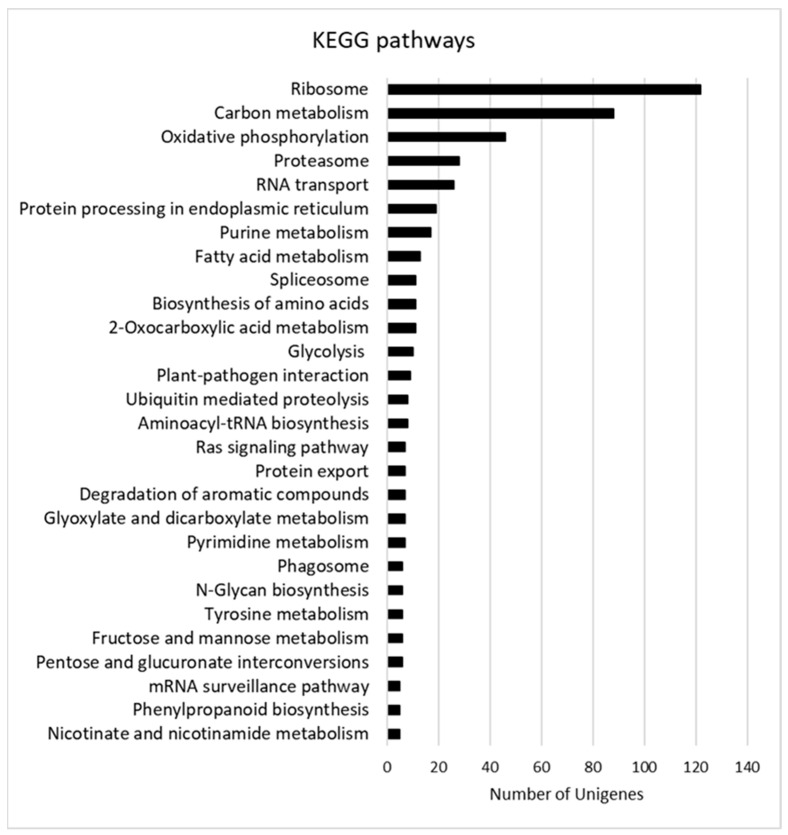
Most represented KEGG pathways. Pathways with at least five Unigenes are listed.

Gene Ontology (GO) terms and the Kyoto Encyclopedia of Genes and Genomes (KEGG) pathway functional enrichments were used to identify possible biological processes or pathways involved in fungus establishment and progression. Considering the GO enrichment, “membrane” (GO:0016020) and “ribonucleoprotein complex” (GO:1990904) are the most enriched terms in the Cellular Component (CC) category. The Molecular Function (MF) category includes mainly “catalytic activity” (GO:0003824), “hydrolase activity” (GO:0016787), “binding” (GO:0005488), and “structural molecule activity” (GO:0005198), whereas “primary metabolic process” (GO:0044238), “oxidation-reduction process” (GO:0055114), “metabolic process” (GO:0008152), and “transport” (GO:0006810) are the main enriched terms in the Biological Process (BP) category (Figure 2). As shown in Figure 3, the main KEGG pathway term is “ribosome” (122), and, although less abundant, the “proteasome” (28) and “ubiquitin-mediated proteolysis” (8), “aminoacyl-tRNA biosynthesis” (8) and “protein export” (7) categories are also represented clearly indicating the occurrence of the protein metabolism remodeling, both biosynthesis, and degradation, during fungus infection. The representation of “carbon metabolism” (88), “oxidative phosphorylation” (46), and “glycolysis” (10) categories suggests that the mitochondrial respiratory chain is highly active during *P. tracheiphilus* infection, this result is expected from a heterotrophic organism.

### 3.4. Classification of Highly Expressed Fungus Genes

**Figure 4:**
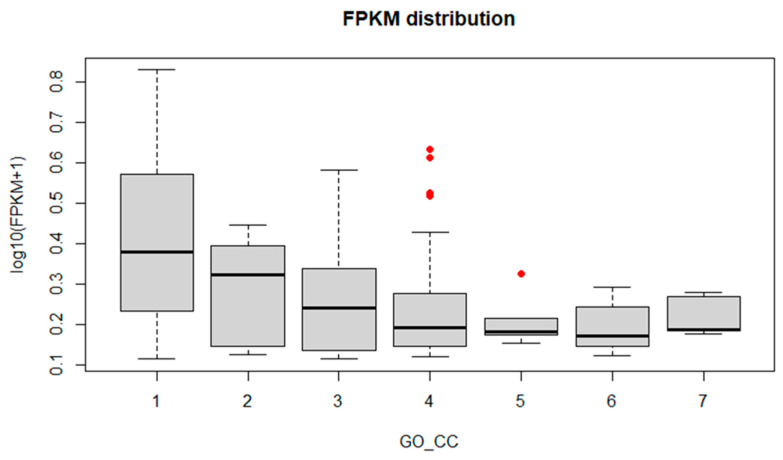
FPKM distribution of the most enriched GO terms (Cellular Component). The *x*-axis corresponds to the enriched terms with more than five Unigenes, the y-axis is the FPKM value expressed as log_10_(FPKM+1) (FPKM > 0.3). Red dots represent the ”outlier” clusters. 1: ribonucleoprotein complex; 2: cell part; 3: membrane part; 4: membrane; 5: proteasome core complex; 6: cytoplasm; 7: cell.

**Figure 5:**
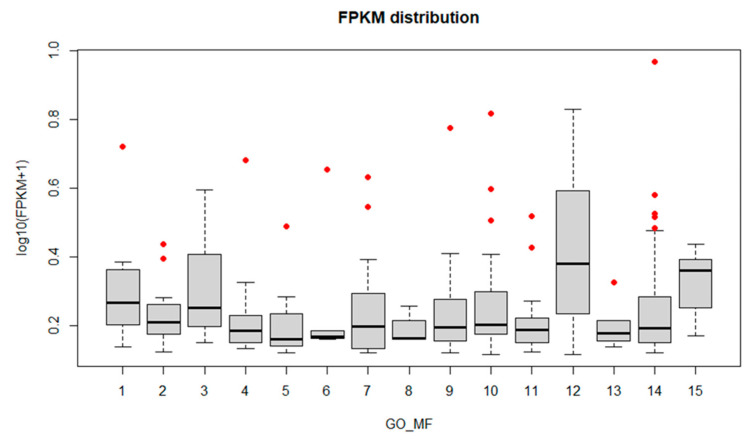
FPKM distribution of the most enriched GO terms (Molecular Function). The x-axis corresponds to the enriched terms with more than five Unigenes, the *y*-axis is the FPKM value expressed as log_10_(FPKM+1) (FPKM > 0.3). Red dots represent the ”outlier” clusters. 1: catalytic activity, acting on a protein; 2: organic cyclic compound binding; 3: coenzyme binding; 4: cation binding; 5: anion binding; 6: small molecule binding; 7: purine ribonucleoside triphosphate binding; 8: heme binding; 9: hydrolase activity; 10: binding; 11: transporter activity; 12: structural molecule activity; 13: endopeptidase activity; 14: catalytic activity; 15: nucleic acid binding.

**Figure 6:**
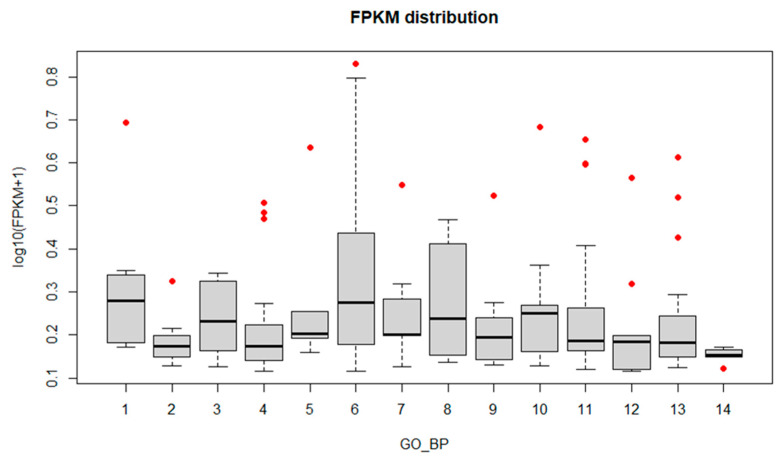
FPKM distribution of the most enriched GO terms (Biological Process). The *x*-axis corresponds to the enriched terms with more than five Unigenes, the *y*-axis is the FPKM value expressed as log_10_(FPKM+1) (FPKM > 0.3). Red dots represent the ”outlier” clusters. 1: proton transmembrane transport; 2: organonitrogen compound metabolic process; 3: organic substance metabolic process; 4: oxidation-reduction process; 5: establishment of localization; 6: primary metabolic process; 7: RNA biosynthetic process; 8: cellular homeostasis; 9: phosphorylation; 10: cellular process; 11: metabolic process; 12: protein localization; 13: transport; 14: nitrogen compound metabolic process.

**Figure 7:**
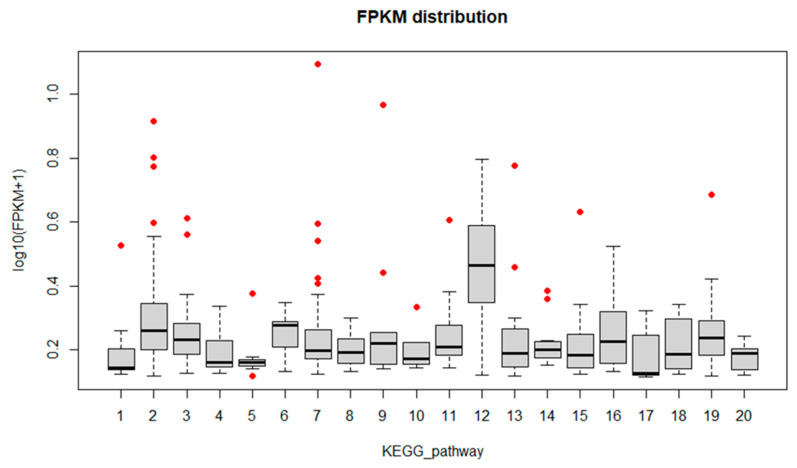
FPKM distribution of the twenty most enriched KEGG pathways. The *x*-axis is the enriched pathway, the *y*-axis is the FPKM value expressed as log_10_(FPKM+1) (FPKM > 0.3). Red dots represent the ”outlier” clusters. 1: glycolysis; 2: oxidative phosphorylation; 3: purine metabolism; 4: pyrimidine metabolism; 5: glyoxylate and dicarboxylate metabolism; 6: aminoacyl-tRNA biosynthesis; 7: carbon metabolism; 8: 2-oxocarboxylic acid metabolism; 9: fatty acid metabolism; 10: degradation of aromatic compounds; 11: biosynthesis of amino acids; 12: ribosome; 13: RNA transport; 14: spliceosome; 15: proteasome; 16: protein export; 17: ras signaling pathway; 18: ubiquitin mediated proteolysis; 19: protein processing in endoplasmic reticulum; 20: plant-pathogen interaction.

To identify fungus genes that might have a crucial role in infection onset and progression, box plot graphs were constructed reporting the log_10_(FPKM+1) values for each GO (Figure 4, Figure 5 and Figure 6) and KEGG categories (Figure 7). These graphs report log_10_(FPKM+1) distribution within each GO term, and the black line is the median value. Concerning the GO terms related to Cellular Component (CC) (Figure 4), the high median value of log_10_(FPKM+1) is registered for the “ribonucleoprotein complex”, followed by “cell part” and “membrane part”. Within the categories “membrane” and “proteasome core complex”, several outlier genes have been identified, with transcripts whose expression [log_10_(FPKM+1)] was outside the fences (“whiskers”) of the boxplot and located above the upper quartile (Figure 4). The boxplot referring to the GO Molecular Function (MF) (Figure 5) revealed a higher median value within the “structural molecule activity” and, more interestingly, several outliers, especially in the “catalytic activity acting on a protein”, “cation binding”, “small molecules binding, “small-molecule binding”, “hydrolase activity”, “binding” and “catalytic activity” (Figure 5). Figure 6 shows the boxplot of the GO Biological Process (BP) terms. In this last case, the median log_10_(FPKM+1) values are similar for all the categories and ranges between 0.1–0.25. Several outliers have been identified in the “proton transmembrane transport”, “oxidation-reduction process, “establishment of localization”, “primary metabolic process”, “RNA biosynthetic process”, “phosphorylation”, “cellular process”, “metabolic process”, “protein localization” and “transport”. Similarly, the log_10_(FPKM+1) distribution within the KEGG categories is displayed in Figure 7. “Ribosome” category has the higher log_10_(FPKM+1) median value indicating that genes belonging to this category are highly expressed by the fungus during infection. Transcripts associated with log_10_(FPKM+1) values above the upper quartile were found in the “oxidative phosphorylation”, “carbon metabolism”, “fatty acid metabolism”, “RNA transport”, “proteasome”, and “protein processing in endoplasmic reticulum” categories. Considering that the higher expression values might be related to a key role in infection onset and progression, all the outlier transcripts (65 outliers in total) are reported in Table S1.

### 3.5. Fungus-Plant Interaction: Identification of Crucial Genes Involved in Fungal Development and Pathogenesis

In the following section, we focused our attention on clusters whose expression values were higher than the FPKM distribution median values on the basis that they might have a major role in pathogenesis [28,29]. Moreover, we paid attention to those clusters annotated in the SwissProt database (33 in total), as this is a rich resource of protein sequences and functional information. 

Table 1 reports the cluster-ID and functional description along with the FPKM values for selected genes. In particular, a “probable GPI-anchored cupredoxin”, coding a soluble electron transfer copper protein involved in the inactivation of copper-containing nitrite reductase in the presence of oxygen, was found highly expressed during the *P. tracheiphilus* infection of rough lemon leaves. 

Protein SnodProt1 homolog was also identified as being a member of the cerato-platanin protein (CPP) family playing a pivotal role in fungal cell wall expansion and in chitin oligomer scavenging, thereby disguising fungal presence and preventing the recognition of fungi by plants [30,31,32]. A transcription factor that confers fluconazole resistance, fluconazole resistance protein 1, and plasma membrane proteolipid 3 (Pmp3p), which seems to increase Amphotericin B (AmB) resistance, were among the retrieved highly expressed fungus transcripts (Table 1) [33,34,35]. Another cluster that seems to be related to the circumvention of plant defense codes catechol 1,2 dioxygenase, catalyzing the cleavage of aromatic rings at the catecholic bond through the addition of molecular oxygen, thus disrupting plant defense compounds [36]. Moreover, endochitinase 1, cell wall mannoprotein PIR3, and cross-pathway control protein 1 (cpcA) were among the expressed fungus transcripts. It has been shown that the encoded proteins are crucial for fungal propagation and pathogenesis, and are responsible for plasticizing the cell wall, specifically during cell separation and in phytotoxin biosynthesis regulation, respectively [37,38,39]. In this respect, the β-cyclopiazonate dehydrogenase encoding gene was among the retrieved sequences. It is part of the gene cluster that mediates the biosynthesis of the fungal toxin cyclopiazonic acid (CPA), a nanomolar inhibitor of Ca^2+^-ATPase and a potent inducer of cell death in plants [40,41]. It oxidizes β-CPA in a two-electron process, subsequently allowing for ring closure and the formation of α-CPA. Finally, the clusters related to the nascent polypeptide-associated complex (NAC), both α and β monomer types, were discovered as sharply expressed fungus transcripts. NAC is a functionally versatile protein complex implicated in protein biogenesis, assembly, and transportation, and it was demonstrated to play a key role in regulating development and pathogenesis [42].

Those clusters that were not among the outlier genes are reported in Table 2, but they are taken into consideration based on their importance in pathogen invasion ability. In particular, several genes encoding fungus pectinesterase, pectin lyase, and endo β-1,4 glucanase are expressed in rough lemon leaves during *P. tracheiplilus* infection, thus confirming the importance of the maceration of plant tissue and the degradation of the complex natural cellulosic layer in pathogenesis [7,8,17]. Interestingly, norsolorinic acid reductase B, the part of the gene cluster that mediates the biosynthesis of the toxic and carcinogenic group of polyketide-derived furanocoumarins known as aflatoxins, was found among the fungus transcripts [43] along with versiconal hemiacetal acetate reductase which is not considered an aflatoxin biosynthesis gene, although it actually participates in its biosynthesis in cells [44] (Table 2). Dehydrogenase patE (glucose methanol choline oxidoreductase, GMC oxidoreductase) catalyzing the terminal step in the biosynthesis of patulin, an acetate-derived tetraketide mycotoxin [45,46], is among the fungus transcripts. These last findings suggest that toxic fungal secondary metabolites that induce several immunological, neurological, and gastrointestinal negative effects in human beings are synthesized during fungus infection. Finally, a group of clusters (trans-enoyl reductase, nonribosomal peptide synthetase, and development regulator fbA) is involved in the production of conidiophore pigment and in the signal transduction pathway that triggers the asexual reproduction, respectively (Table 2).

## 4. Discussion

The application of Next Generation Sequencing techniques in RNA sequencing simplifies the performance of functional genomic studies to understand the plant response to both biotic and abiotic stress [23,25,47]. Moreover, RNA sequencing and de novo assembly of the clean reads have become a feasible method to define the transcriptome even in species whose genome has not yet been sequenced. Previously, we dissected the transcriptomic profile of rough lemon leaves 15 days after artificial inoculation with *P. tracheiphilus*, this being a fully compatible host-pathogen interaction [23]. 

The aim of the current work was to look at “the other side of the coin” in order to retrieve and classify the fungus transcriptome expressed during rough lemon infection and decipher the potential mechanisms and processes that the fungus carries out to manifest pathogenesis. This approach was applied to identify *B. sorghicola* transcripts that were expressed during the growth of the fungus in sorghum [48], provide insights into the molecular and genetic basis of *Pyrenochaeta lycopersici* lifestyle by characterizing previously unknown pathogenic behaviors in tomato [49], and identify genes that the pathogen uses to avoid host detection or to derive nutrition from it [50]. More recently, simultaneous RNAseq in combination with de novo assembly in rice during the necrotrophic phase of *Rhizoctonia solani* led to the conclusion that pathogenesis depends on the capacity to damage the host cell wall and to manage oxidative stress and cytotoxic compounds [51]. Similarly, during the infection of *Physcomitrium patens*, the fungus *Botrytis cinerea* upregulated genes involved in reactive oxygen species generation and detoxification, transporter activities, plant cell wall degradation and modification, toxin production, and possible plant defense evasion by effector proteins [52]. 

As aforementioned, the following discussion is focused on clusters whose expression values were higher than the FPKM distribution median values on the basis that they might have a major role in pathogenesis [28,29]. According to this assessment, we were able to identify pathways and metabolisms potentially involved in the fungus infection establishment and progression. The strategies brought about are related to (a) fungus cell wall remodeling and protection, (b) destroying plant defensive secondary metabolites, (c) optimizing fungus development and pathogenesis, and (d) toxin biosynthesis, all of these confirm previously obtained results from recent decades [4,7,8,17,21,53]. Endochitinases, cell wall mannoprotein PIR3, and SnodProt1 (cerato-platanin protein, CPP) play key roles in the aforementioned first strategy. The cell wall is a dynamic organelle with great adaptive flexibility that allows remodeling, morphogenesis, and changes in its components in response to the environment. It is mainly composed of the inner polysaccharide-rich layer (chitin and β-glucan) and the outer protein coat (mannoproteins). Endochitinases are involved in the early events of host–parasite interactions of biotrophic and necrotrophic mycoparasites and entomopathogenic fungi, as well as vesicular-arbuscular mycorrhizal fungi [54]. They can have a housekeeping function in plasticizing the fungus cell wall or can act more specifically during cell separation and nutritional chitin acquisition. Moreover, chitin polymers and their modified form, chitosan, induce host defense response through the MAMP-triggered immunity (MTI) [54]. Cell wall mannoprotein PIR3 is a component of the outer cell wall layer and is required for its stability. The mannoproteins are also associated with adherence, drug resistance, and virulence [39]. SnodProt1 (cerato-platanin protein, CPP) was firstly identified from the phytotoxic wheat pathogen *Stagonospora nodorum* [55]. Although the specific mechanism by which CPPs contribute to virulence is still unknown, a clear involvement in virulence has been demonstrated in cotton infected by *V. dahliae*, which, similarly to *P. tracheiphilus*, is a xylem-colonizing fungus [56]. Recombinant *Vd*CP1 protein induces local and systemic defense responses in host plants and protects the *V. dahliae* cell wall from enzymatic degradation, probably related to its chitin-binding and protection properties [56]. 

Among the transcripts involved in destroying plant or external secondary metabolites, the function of the Pmp3/Sna family has not yet been resolved. Amphotericin B (AmB) is one of the few antifungals available to treat invasive opportunistic fungal infections in humans [34]. AmB is currently considered to kill fungi by forming a large, extramembranous fungicidal sterol sponge that depletes ergosterol from lipid bilayers. Recently, overexpression of PMP3 gene was shown to increase AmB resistance that requires a functional sphingolipid pathway, suggesting that Pmp3p could be part of a phosphoinositide-regulated stress sensor [35]. A second way to avoid plant or external defense molecules seems to occur during *P. tracheiplilus* infection, as suggested by the expression of fluconazole resistance protein 1 which is a transcription factor that confers fluconazole resistance in *S. cerevisiae* by activation of the PDR5 gene, a gene coding for a drug efflux transporter of the ATP-binding cassette superfamily [33]. Fluconazole works by inhibiting 14α-demethylase, a key enzyme involved in ergosterol synthesis. By inhibiting this enzyme, there is an accumulation of ergosterol precursors within the fungal cell that, when they reach high levels, become toxic and cause alterations in the permeability of the cell membrane. Similarly, as observed for some fungi [36], catechol 1,2 dioxygenase might intervene to overcome host defense responses through the cleavage of the benzene ring of catechol, the main intermediate in the degradation of plant defensive aromatic compounds. 

Obviously, an accurate balance among protein biosynthesis, assembly, and transport is a prerequisite for a successful host invasion. In this respect, the nascent polypeptide-associated complex (NAC) is a functionally versatile protein complex that is involved in protein homeostasis (proteostasis), regulating the development and pathogenesis of *F. graminearum* in wheat [42]. The last group of outlier genes comprises those involved in toxin biosynthesis (the cross-pathway control genes, cpcA, and β-cyclopiazonate dehydrogenase). Several extracellular lipophilic and hydrophilic phytotoxic compounds have been isolated from the culture filtrates of the *P. tracheiphilus*, leading to a wide range of molecular weight compounds [57,58,59,60,61,62]. These findings validate the hypothesis that the toxins have an essential role in the etiology of mal secco [8]. In 1979, Nachmias and co-authors [63] showed that the toxic compound was a complex of glycoproteins, which was named malseccin. These toxins had an estimated molecular weight of 60 and 93 kDa, and it was demonstrated that both glycoproteins could emulate the typical symptoms of MSD-like leaf vein chlorosis and necrosis [19,21]. However, a significant phytotoxic fraction was identified in the glycopeptide of 60 kDa, called Pt60. In our work, β-cyclopiazonate dehydrogenase was highly expressed in rough lemon infected leaves, suggesting that a toxin structurally similar to the fungal neurotoxin cyclopiazonic acid (CPA) [64] might be involved in *P. tracheiphilus* pathogenesis. CPA inhibits the pumping of Ca^2+^ into cellular compartments leading to an uncontrolled rise of cytosolic calcium, provoking plant cell death [41]. β-cyclopiazonate dehydrogenase is not the enzyme deducible from the protein sequenced by Fogliano et al. (1998) [21], thus suggesting that an array of toxic compounds of both high and low molecular weight could be produced by *P. tracheiphilus* infection. The identification of a low molecular weight and heat stable (100 °C, 15 min) toxic compound in *P. tracheiphilus* during growth on a synthetic medium corroborates this hypothesis [57]. Furthermore, the finding of the highly expressed cross-pathway control gene (cpcA), a homolog of the plant pathogen *Leptosphaeria maculans* (Desm.), having a crucial role in the production of the major phytotoxin [37] suggests that a similar toxin might be produced by *P. tracheiplilus*. 

Moreover, aflatoxins and patulin biosynthetic genes have been found among the *P. tracheiphilus* transcripts. These secondary metabolites produced by certain molds, particularly by *Aspergillus* and *Penicillium* species, represent a threat to human health in contaminated food. It has been shown that aflatoxins can retard plant growth, but there is no evidence for their involvement in pathogenesis [41]. Finally, the discovery among the non-outliers (Table 2) of several genes encoding fungal enzymes devoted to plant cell wall degradation is in accordance with several previous works indicating that the production of hydrolytic enzymes and the subsequent release of nutrient sources have a substantial role in virulence [7,8,17].

## 5. Conclusions

In conclusion, the analysis of the *P. tracheiphilus* transcriptome while infecting rough lemon leaves led to the identification of highly expressed genes mainly distributed in four specific groups involved in (a) fungus cell wall remodeling and protection, (b) destroying plant defensive secondary metabolites, (c) optimizing fungus development and pathogenesis, and (d) toxin biosynthesis, thus revealing a multifaceted strategy to make the host succumb. Our results may be relevant to help in identifying sustainable field treatments to cope with disease diffusion and to provide direction into possible biotechnological approaches to generate resistant lemon plants.

## Figures and Tables

**Table 1:** Outlier fungus transcripts expressed during rough lemon infection.

Cluster ID	Database Description	Percent Identity	Evalue	FPKM
17189.0	GPI-anchored cupredoxin ARB SwissProt: P0DN32 (*P. tracheiphilus*), Sequence ID: KAF2852624.1	100%	4 × 10^−89^	9.374
16536.0	Protein SnodProt1 SwissProt: O74238 (*Parastagonospora nodorum*), Sequence ID: O74238.1	79%	3 × 10^−75^	8.880
1627.0	Glucose-repressible gene SwissProt: P22151 (*Akanthomyces lecanii* RCEF 1005), Sequence ID: OAA78938.1	85%	1 × 10^−33^	5.533
14701.25619	Plasma membrane proteolipid 3 SwissProt: Q4HXT6 (*Stemphylium lycopersici*), Sequence ID: KNG45495.1	98%	3 × 10^−30^	3.663
15912.0	Endochitinase B1 SwissProt: Q873X9 (*Leptosphaeria biglobosa*), SequenceID: KAH9877693.1	78%	0.0	2.660
4528.0	Cross-pathway control protein 1 SwissProt: P87090 (*Alternaria alternata*), Sequence ID: KAH8629237.1	67%	5 × 10^−148^	2.079
18021.0	Catechol 1,2-dioxygenase SwissProt: P86029 (*Alternaria panax*), Sequence ID: KAG9186190.1	94%	0.0	1.602
12526.0	Cell wall mannoprotein PIR3 SwissProt: A6ZZG1 (*Plenodomus tracheiphilus*), Sequence ID: KAF2851031.1	100%	2 × 10^−170^	1.411
20096.0	Beta-cyclopiazonate dehydrogenase SwissProt: Q2UG11 (*Colletotrichum truncatum*), Sequence ID: XP_036575998.1	62%	0.0	1.320
14701.7227	Fluconazole resistance protein 1 C6 transcription factor-like protein SwissProt: O93870 (*Plenodomus tracheiphilus*), Sequence ID: AF057038.1	99%	0.0	1.320
17612.0	Nascent polypeptide associated complex subunit beta SwissProt: Q0ULD0 (*Parastagonospora nodorum*), Sequence ID: Q0ULD0.1	92%	2 × 10^−91^	1.127
17977.0	Nascent polypeptide associated complex subunit alpha SwissProt: Q0UKB5 (*Plenodomus tracheiphilus*), Sequence ID: KAF2851025.1	100%	4 × 10^−58^	1.113

**Table 2:** Non-outlier fungus transcripts expressed during rough lemon infection.

Cluster ID	Database Description	Percent Identity	Evalue	FPKM
21106.0	Pectinesterase [*Alternaria arborescens*]	74.03%	1 × 10^−157^	1.725
6634.0	Pectin lyase-like protein [*Setomelanomma holmii*]	88.16%	0.0	0.700
8430.0	pectate lyase-like protein [*Alternaria alternata*]	82.62%	1 × 10^−173^	0.479
19444.0	Putative endo-beta-1,4-glucanase D [*Leotiomycetes* sp. MPI-SDFR-AT-0126]	73.08%	3 × 10^−120^	0.413
15121.0	GMC oxidoreductase [*Plenodomus tracheiphilus* IPT5]	100.00%	2 × 10^−69^	0.637
7879.0	Norsolorinic acid reductase A [*Alternaria arborescens*]	88.00%	0.0	0.706
22064.0	Versiconal hemiacetal acetate reductase [*Alternaria arborescens*]	87.00%	0.0	0.322
12949.0	zinc-binding oxidoreductase-like protein ToxD [*Plenodomus tracheiphilus* IPT5] Trans-enoyl reductase fsr4	100%	0.0	0.469
11498.0	Di-copper centre-containing protein [*Plenodomus tracheiphilus* IPT5] N-acetyl-6-hydroxytryptophan oxidase ivoB	100%	0.0	0.357
8092.0	Developmental regulator flbA [*Alternaria gaisen*] KAB2108722.1	89%	6 × 10^−102^	0.683

## Data Availability

The data presented in this study are openly available in NCBI (https://www.ncbi.nlm.nih.gov/geo/, reference number GSE164096, accessed on 29 December 2020).

## Supplementary Materials

The following supporting information can be downloaded at: https://www.mdpi.com/article/10.3390/biology11050761/s1, Table S1: Outlier gene list.

## References

[1] de Gruyter H., Woudenberg J.H.C., Aveskamp M.M., Verkley G.J.M., Groenewald J.Z., Crous P.W. (2013). Redisposition of phoma-like anamorphs in pleosporales. Stud. Mycol..

[2] Ben-Hamo M., Ezra D., Krasnov H., Blank L. (2020). Spatial and temporal dynamics of Mal Secco disease spread in lemon orchards in Israel. Phytopathology.

[3] Catalano C., Di Guardo M., Distefano G., Caruso M., Nicolosi E., Deng Z., Gentile A., La Malfa S.G. (2021). Biotechnological Approaches for Genetic Improvement of Lemon (*Citrus limon* (L.) Burm. f.) against Mal Secco Disease. Plants.

[4] Catara A., Catara V. (2019). Il “mal secco” degli agrumi, da un secolo in Sicilia. Memorie e Rendiconti.

[5] Solel Z. (1976). Epidemiology of Mai Secco Disease of Lemons. J. Phytopathol..

[6] Cutuli G., Salerno M. On the epidemiological meaning of phialospores in Phoma tracheiphila (Petri) Kanc. et Ghik. Proceedings of the 5th Congress of the Mediterranean Phytopathological Union.

[7] Nigro F., Ippolito A., Salerno M.G. (2011). Mal secco disease of citrus: A journey through a century of research. J. Plant Pathol..

[8] Migheli Q., Cacciola S.O., Balmas V., Pane A., Ezra D., Di San Lio G.M. (2009). Mal Secco Disease Caused by Phoma tracheiphila: A Potential Threat to Lemon Production Worldwide. Plant Dis..

[9] Deb D., Khan A., Dey N. (2020). Phoma diseases: Epidemiology and control. Plant Pathol..

[10] Magnano Di San Lio G., Lo Giudice L. (1982). Role of cell wall in gum production in “Citrus”. Caryologia.

[11] Balmas V., Scherm B., Ghignone S., Salem A.O.M., Cacciola S.O., Migheli Q. (2005). Characterisation of Phoma tracheiphila by RAPD-PCR, microsatellite-primed PCR and ITS rDNA sequencing and development of specific primers for in planta PCR detection. Eur. J. Plant Pathol..

[12] Grasso F.M., Catara V. (2006). Preliminary characterization of Phoma tracheiphila isolates from Italy and Greece by DNA-based typing methods. J. Plant Pathol..

[13] Licciardello G., Grasso F.M., Bella P., Cirvilleri G., Grimaldi V., Catara V. (2006). Identification and detection of Phoma tracheiphila, causal agent of citrus mal secco disease, by real-time polymerase chain reaction. Plant Dis..

[14] Ezra D., Kroitor T., Sadowsky A. (2007). Molecular characterization of Phoma tracheiphila, causal agent of Mal secco disease of citrus, in Israel. Eur. J. Plant Pathol..

[15] Russo M., Grasso F.M., Bella P., Licciardello G., Catara A., Catara V. (2011). Molecular diagnostic tools for the detection and characterization of Phoma tracheiphila. Acta Hortic..

[16] Magnano Di San Lio G., Perrotta G., Cavalloro R., Di Martino E. (1986). Variabilità in Phoma tracheiphila. Integrated Pest Control in Citrus Groves.

[17] Cacciola S.O., Natoli M., Pane A., Perrotta G., Petrone G. (1990). Characterization of polygalacturonase activities from Phoma tracheiphila. Ital. J. Biochem..

[18] Shao D., Smith D.L., Kabbage M., Roth M.G. (2021). Effectors of Plant Necrotrophic Fungi. Front. Plant Sci..

[19] Nachmias A., Barash I., Solel Z., Strobel G.A. (1977). Purification and characterization of a phytotoxin produced by Phoma tracheiphila, the causal agent of mal secco disease of citrus. Physiol. Plant Pathol..

[20] Gentile A., Tribulato E., Continella G., Vardi A. (1992). Differential responses of citrus calli and protoplasts to culture filtrate and toxin of Phoma tracheiphila. Theor. Appl. Genet..

[21] Fogliano V., Marchese A., Scaloni A., Ritieni A., Visconti A., Randazzo G., Graniti A. (1998). Characterization of a 60 kDa phytotoxic glycoprotein produced by Phoma tracheiphila and its relation to malseccin. Physiol. Mol. Plant Pathol..

[22] Russo R., Caruso M., Arlotta C., Piero A.R.L., Nicolosi E., Di Silvestro S. (2020). Identification of field tolerance and resistance to mal secco disease in a citrus germplasm collection in sicily. Agronomy.

[23] Russo R., Sicilia A., Caruso M., Arlotta C., Di Silvestro S., Gmitter F.G., Nicolosi E., Lo Piero A.R. (2021). De novo transcriptome sequencing of rough lemon leaves (*Citrus jambhiri* Lush.) in response to Plenodomus tracheiphilus infection. Int. J. Mol. Sci..

[24] Sicilia A., Testa G., Santoro D.F., Cosentino S.L., Lo Piero A.R. (2019). RNASeq analysis of giant cane reveals the leaf transcriptome dynamics under long-term salt stress. BMC Plant Biol..

[25] Sicilia A., Santoro D.F., Testa G., Cosentino S.L., Lo Piero A.R. (2020). Transcriptional response of giant reed (*Arundo donax* L.) low ecotype to long-term salt stress by unigene-based RNAseq. Phytochemistry.

[26] Grasso F.M. (2008). Caratterizzazione Fenotipica di Isolati del Fungo Phoma Tracheiphila e Sviluppo di un Metodo di Rilevamento Quantitativo Mediante Real-Time PCR.

[27] Salerno M., Catara V. (1967). Ricerche sul “mal secco” degli Agrumi (Deutero-phoma tracheiphila Petri). IV. Comportamento parassitario del fungo in ospiti diversi dagli Agrumi. Tec. Agric..

[28] Hart T., Komori H.K., LaMere S., Podshivalova K., Salomon D.R. (2013). Finding the active genes in deep RNA-seq gene expression studies. BMC Genom..

[29] Hebenstreit D., Fang M., Gu M., Charoensawan V., Van Oudenaarden A., Teichmann S.A. (2011). RNA sequencing reveals two major classes of gene expression levels in metazoan cells. Mol. Syst. Biol..

[30] Baccelli I. (2015). Cerato-platanin family proteins: One function for multiple biological roles?. Front. Plant Sci..

[31] Frischmann A., Neudl S., Gaderer R., Bonazza K., Zach S., Gruber S., Spadiut O., Friedbacher G., Grothe H., Seidl-Seiboth V. (2013). Self-assembly at air/water interfaces and carbohydrate binding properties of the small secreted protein EPL1 from the fungus Trichoderma atroviride. J. Biol. Chem..

[32] Gaderer R., Bonazza K., Seidl-Seiboth V. (2014). Cerato-platanins: A fungal protein family with intriguing properties and application potential. Appl. Microbiol. Biotechnol..

[33] Talibi D., Raymond M. (1999). Isolation of a Putative Candida albicans Transcriptional Regulator Involved in Pleiotropic Drug Resistance by Functional Complementation of a pdr1 pdr3 Mutation in Saccharomyces cerevisiae. J. Bacteriol..

[34] Bari V.K., Sharma S., Alfatah M., Mondal A.K., Ganesan K. (2015). Plasma Membrane Proteolipid 3 Protein Modulates Amphotericin B Resistance throughSphingolipid Biosynthetic Pathway. Sci. Rep..

[35] de Block J., Szopinska A., Guerriat B., Dodzian J., Villers J., Hochstenbach J.F., Morsomme P. (2015). Yeast Pmp3p has an important role in plasma membrane organization. J. Cell Sci..

[36] Soal N.C., Coetzee M.P.A., van der Nest M.A., Hammerbacher A., Wingfield B.D. (2022). Phenolic degradation by catechol dioxygenases is associated with pathogenic fungi with a necrotrophic lifestyle in the Ceratocystidaceae. G3 Genes|Genome|Genet..

[37] Elliott C.E., Fox E.M., Jarvis R.S., Howlett B.J. (2011). The cross-pathway control system regulates production of the secondary metabolite toxin, sirodesmin PL, in the ascomycete, Leptosphaeria maculans. BMC Microbiol..

[38] Langner T., Göhre V. (2016). Fungal chitinases: Function, regulation, and potential roles in plant/pathogen interactions. Curr. Genet..

[39] Ibe C., Munro C.A. (2021). Fungal Cell Wall Proteins and Signaling Pathways Form a Cytoprotective Network to Combat Stresses. J. Fungi.

[40] Liu X., Walsh C.T. (2009). Cyclopiazonic acid biosynthesis in *Aspergillus* sp.: Characterization of a reductase-like R* domain in cyclopiazonate synthetase that forms and releases cyclo-acetoacetyl-L-tryptophan. Biochemistry.

[41] Chalivendra S.C., De Robertis C., Chang P.K., Damann K.E. (2017). Cyclopiazonic acid is a pathogenicity factor for *Aspergillus flavus* and a promising target for screening germplasm for ear rot resistance. Mol. Plant-Microbe Interact..

[42] Wang X., Xie X., Liu J., Wang G.L., Qiu D. (2020). Nascent Polypeptide-Associated Complex Involved in the Development and Pathogenesis of Fusarium graminearum on Wheat. Engineering.

[43] Yu J., Bhatnagar D., Cleveland T.E. (2004). Completed sequence of aflatoxin pathway gene cluster in *Aspergillus parasiticus*. FEBS Lett..

[44] Shima Y., Shiina M., Shinozawa T., Ito Y., Nakajima H., Adachi Y., Yabe K. (2009). Participation in aflatoxin biosynthesis by a reductase enzyme encoded by vrdA gene outside the aflatoxin gene cluster. Fungal Genet. Biol..

[45] Artigot M.P., Loiseau N., Laffitte J., Mas-Reguieg L., Tadrist S., Oswald I.P., Puel O. (2009). Molecular cloning and functional characterization of two CYP619 cytochrome P450s involved in biosynthesis of patulin in *Aspergillus clavatus*. Microbiology.

[46] Tragni V., Cotugno P., De Grassi A., Cavalluzzi M.M., Mincuzzi A., Lentini G., Sanzani S.M., Ippolito A., Pierro C.L. (2021). Targeting Penicillium expansum GMC Oxidoreductase with High Affinity Small Molecules for Reducing Patulin Production. Biology.

[47] Fiore M.C., Mercati F., Spina A., Blangiforti S., Venora G., Dell’Acqua M., Sunseri F. (2019). High-Throughput Genotype, Morphology, and Quality Traits Evaluation for the Assessment of Genetic Diversity of Wheat Landraces from Sicily. Plants.

[48] Yazawa T., Kawahigashi H., Matsumoto T., Mizuno H. (2013). Simultaneous Transcriptome Analysis of Sorghum and Bipolaris sorghicola by Using RNA-seq in Combination with De Novo Transcriptome Assembly. PLoS ONE.

[49] Aragona M., Minio A., Ferrarini A., Valente M.T., Bagnaresi P., Orrù L., Tononi P., Zamperin G., Infantino A., Valè G. (2014). De novo genome assembly of the soil-borne fungus and tomato pathogen Pyrenochaeta lycopersici. BMC Genom..

[50] Lowe R.G.T., Cassin A., Grandaubert J., Clark B.L., Van De Wouw A.P., Rouxel T., Howlett B.J. (2014). Genomes and transcriptomes of partners in plant-fungal- interactions between canola (*Brassica napus*) and two *Leptosphaeria* species. PLoS ONE.

[51] Ghosh S., Kanwar P., Jha G. (2018). Identification of candidate pathogenicity determinants of Rhizoctonia solani AG1-IA, which causes sheath blight disease in rice. Curr. Genet..

[52] Reboledo G., Agorio A., Vignale L., Batista-García R.A., Ponce De León I. (2021). Botrytis cinerea transcriptome during the infection process of the bryophyte physcomitrium patens and angiosperms. J. Fungi.

[53] Strano C.P., Bella P., Licciardello G., Fiore A., Lo Piero A.R., Fogliano V., Venturi V., Catara V. (2015). Pseudomonas corrugata crpCDE is part of the cyclic lipopeptide corpeptin biosynthetic gene cluster and is involved in bacterial virulence in tomato and in hypersensitive response in Nicotiana benthamiana. Mol. Plant Pathol..

[54] Pusztahelyi T. (2018). Chitin and chitin-related compounds in plant-fungal interactions. Mycology.

[55] Skinner W., Keon J., Hargreaves J. (2001). Gene information for fungal plant pathogens from expressed sequences. Curr. Opin. Microbiol..

[56] Zhang Y., Gao Y., Liang Y., Dong Y., Yang X., Yuan J., Qiu D. (2017). The verticillium dahliae snodprot1-like protein VdCP1 contributes to virulence and triggers the plant immune system. Front. Plant Sci..

[57] Barash I., Pupkin G., Koren L., Ben-Hayyim G., Strobel G.A. (1981). A low molecular weight phytotoxin produced by Phoma tracheiphila, the cause of mal secco disease in citrus. Physiol. Plant Pathol..

[58] Graniti A. Host-parasite relations in citrus diseases as exemplified by Phytophthora gummosis and Deuterophoma mal secco. Proceedings of the 1st International Citrus Symposium.

[59] Parisi A., Tringali C., Magnano Di San Lio G., Cacciola S.O. Phytotoxic activity of mellein; a low-molecular weight metabolite of Phoma tracheiphila. Proceedings of the International Society of Citriculture.

[60] Parisi A., Piattelli M., Tringali C., Di San Lio G.M. (1993). Identification of the phytotoxin mellein in culture fluids of Phoma tracheiphila. Phytochemistry.

[61] Pennisi A.M., Di Pasquale G., Bonforte M., Sesto F., Goren R., Mendel K. (1988). Phytotoxic metabolites of ipo-virulent Phoma tracheiphila isolates. Citriculture, Proceedings of the Sixth International Citrus Congress, Tel Aviv, Israel, 6–11 March, 1988.

[62] Tringali C., Parisi A., Piattelli M., Magnano Di San Lio G. (1993). Phomenin A and B, bioactive polypropionate pyrones from culture fluids of Phoma tracheiphila. Nat. Prod. Lett..

[63] Nachmias A., Barash I., Buchner V., Solel Z., Strobel G.A. (1979). A phytotoxic glycopeptide from lemon leaves infected with Phoma fracheiphila. Physiol. Plant Pathol..

[64] Kato N., Tokuoka M., Shinohara Y., Kawatani M., Uramoto M., Seshime Y., Fujii I., Kitamoto K., Takahashi T., Takahashi S. (2011). Genetic Safeguard against Mycotoxin Cyclopiazonic Acid Production in *Aspergillus oryzae*. ChemBioChem.

